# Influence of Growth Parameters on the Formation of Hydroxyapatite (HAp) Nanostructures and Their Cell Viability Studies

**DOI:** 10.5772/60116

**Published:** 2015-01-01

**Authors:** Murugesan Manoj, Ramesh Subbiah, Devanesan Mangalaraj, Nagamony Ponpandian, Chinnuswamy Viswanathan, Kwideok Park

**Affiliations:** 1 Department of Nanoscience and Technology, Bharathiar University, Tamil Nadu, India; 2 Center for Biomaterials, Korea Institute of Science and Technology (KIST), Seoul, Korea; 3 Department of Biomedical Engineering, Korea University of Science and Technology, Daejeon, Korea

**Keywords:** Hydroxyapatite, β-TCP, nanoparticles, chemical method, biocompatibility

## Abstract

Morphology controlled hydroxyapatite (HAp) nanostructures play a vital role in biomedical engineering, tissue regenerative medicine, biosensors, chemotherapeutic applications, environmental remediation, etc. The present work investigates the influence of temperature, pH and time on the growth of HAp nanostructures using a simple, cost effective and surfactant free chemical approach. The obtained HAp nanostructures were systematically investigated by analytical techniques such as XRD, FESEM, EDX, FTIR and Raman spectroscopy. The XRD analysis showed that the hexagonal structure of the hydroxyapatite and average crystallite size was estimated from this analysis. The electron microscopic analysis confirmed the different morphologies obtained by varying the synthesis parameters such as temperature, pH and time. The elemental composition was determined through EDS analysis. FTIR and Raman spectroscopic analysis confirmed the presence of functional groups and the purity and crystallinity of the samples. The biocompatibility and adhesion nature of samples was examined with mouse preosteoblast cells. The obtained results demonstrated good biocompatibility and excellent focal adhesion.

## 1. Introduction

Hydroxyapatite (HAp)-[Ca_10_(PO_4_)_6_(OH)_2_] is a calcium phosphate ceramic mineral present in the human body. It has for many decades been used as a key material in biomedical engineering as bone implants and prosthesis for dental and bone repair [1]. Biomaterials with nanostructures have unique physicochemical properties and many nano-rooms for the functionalization of biomolecules, and to act as carriers for therapeutics [2]. Morphologically controlled hydroxyapatite nanostructures fulfil multifunctional roles, e.g., in the form of biosensors [3], photocatalysts [4, 5], carriers for drug delivery [6], appliances in tissue engineering and regenerative medicine [7], as well as membranes for the removal of heavy metals from polluted water [8]. These functional properties of HAp strongly depend on their morphology, stoichiometric ratio, crystallinity and crystal size distribution. However, acquiring the desired morphology by controlling the synthesis parameters is difficult and it is a challenge to attain the desired morphology with tuneable properties. Hence, researchers have focused on producing tuneable HAp nanostructures with well-distributed and high crystalline nanostructures.

Nanostructured HAp has been prepared using several methods, e.g., hydrothermal [9, 10], high gravity [11], sol-gel [10], solid state reaction [12], microwave [13, 14], reflux condensation [15], ultra sonication [16, 17], green synthesis [18, 19], mechano-chemical [20, 21], micro emulsion [22, 23] and complexing agent-assisted precipitation [24–26] techniques. In all of these methods, the tweaking of parameters for achieving the desired morphology and good crystalline nanostructures is not easy. Furthermore, among all these methods, aqueous precipitation is the most suitable for tweaking parameters at a low cost. Previous reports have demonstrated the effect of varying temperature during the precipitation reaction of calcium hydroxide (Ca(OH)_2_) and ortho-phosphoric acid (H_3_PO_4_), wherein the spherical HAp nanoparticles formed only at 100°C, while needle-like HAp structures formed at a lower temperature of 40°C [27]. Other researchers have utilized both precipitation and sol-gel processes at 90°C with two hours stirring, wherein Ca(OH)_2_/H_3_PO_4_ and calcium nitrate tetrahydrate/ diammonium hydrogen phosphate (Ca(NO_3_)_2_.4H_2_O/ (NH_4_)_2_HPO_4_) were used as starting precursors [28].

Starting materials also play an important role in producing the desired morphology and crystallinity, and as such, appropriate selection is essential. The prepared powders sometimes exhibit a highly amorphous phase and can be transformed to a crystalline phase by heating for a period of time. Another study demonstrated that the effect of temperature and the mode used for the addition of reactants affected the growth of HAp when using the precipitation method. Nanocrystalline spherical HAp powder could be formed at 40°C using the fast addition mode and rod-like HAp could be formed at 80°C using an ethanol-water mixture as a solvent [29]. It was also reported [30] that changing the temperature and stirring time (24 h) with Ca(OH)_2_ and H_3_PO_4_ as starting reagents produced higher crystallinity. The effect of pH on the precipitation of HAp on silica gels was investigated through complex solutions using the reflux method [12]. It was reported that the change in pH influenced the formation of different morphological shapes and sizes, e.g., needle, fibre, bars, etc.

Most of the previous reports deal with any one of the parameters, such as temperature or pH, or time or the mode of addition of precursors (calcium and phosphate ion source) for the formation of HAp nanostructures [12, 27–30] via different methodologies [9–26]. In the present study, we focused our investigation on the influence of temperature, time and pH on the formation of pristine HAp nanostructures through a simple, cost effective and surfactant free chemical method, using calcium chloride and diammonium hydrogen phosphate as starting precursors. We also studied the cell viability and proliferation of the prepared HAp nanostructures using mouse preosteoblasts.

## 2. Experimental procedure

### 2.1 Materials and equipment

Calcium chloride dihydrate (CaCl_2_.2H_2_O) and diammonium hydrogen phosphate ((NH_4_)_2_HPO_4_) obtained from HiMedia Laboratories Pvt. Ltd. India were used as calcium and phosphate ion sources, respectively. An ammonium solution supplied by SD-Fine Chem Ltd. India was used to adjust the pH of the solutions.

The Bruker D8 powder X-ray diffractometer with Ni filtered Cu-Kα radiation was used to obtain the X-ray diffraction pattern. Continuous scans were done over the 2 theta range of 20° to 80° and with a step size of 0.03°. A FEG Quanta-250 field emission scanning electron microscope was used to obtain the morphology of the prepared sample with an operating voltage of 30 kV. A Jasco FTIR spectrometer and micro Raman spectrometer (LabRAM HR, output power 20 mW with 514 nm argon laser, Horiba Jobin Yvon) were used to record FTIR and Raman spectra.

### 2.2 Synthesis and methodology

The surfactant-free hydroxyapatite nanostructures were prepared using the simple chemical precipitation method. In a typical process, calcium chloride dihydrate (CaCl_2_.2H_2_O) and diammonium hydrogen phosphate ((NH_4_)_2_HPO_4_) were used as starting precursor materials. Calcium chloride and diammonium hydrogen phosphate were mixed separately using double distilled water and with a molar ratio of 1:0.6 to maintain the stoichiometric ratio of hydroxyapatite at 1.67. The pH of the phosphate containing solution was raised to 11 by adding ammonia (25%). Then, the solution was stirred for 1 h and the calcium ion containing solution was added to the phosphate ion containing solution. A jelly-like white precipitate mixture was formed and was stirred for 24 h at room temperature. The final product was washed with distilled water and dried in a hot air oven at 100°C for 24 h. The same procedure was repeated with different temperature conditions, attained by using an ice bath at 0°C and a heating mantle for higher temperatures (60°C and 90°C). Similar reactions were carried out for different stirring periods such as 24, 48 and 72 h, as well as different pH conditions such as 7, 9 and 11.

### 2.3 In-vitro cell viability and proliferation

Mouse preosteoblasts-E1, procured from the Korea Cell Line Bank, were used for the in-vitro study to examine cell viability and proliferation of the prepared HAp nanostructures. For the CCK-8 assay, on day one, 1.0×10^4^ cells were placed in each well of a 96-well plate in 100 μL of an α-modified Eagle's medium containing 10% foetal bovine serum (FBS) and cultured for 12 h at 37°C. 50-μL Aliquot of the colloidal solution of materials in PBS (pH 7.0) was added to the cell culture plate and incubated for one day. The final concentration of the HAp samples ranged from 16×10^−3^ to 1.024 mg/mL. The proliferation of preosteoblasts was evaluated in triplicate after the transfection using a colorimetric CCK-8 assay kit (Dojindo Molecular Technologies Inc., Gaithersburg, MD, USA) and according to the manufacturer's protocols. Measurement was performed using a Magellan ELISA microplate reading spectrophotometer (Tecan) at a wavelength of 450 nm. Relative viability was calculated using 0% (wells without cells) and 100% (wells with untreated cells) controls. Furthermore, a live/dead cell viability assay (Invitrogen) was performed according to the manufacturer's instructions. Briefly, 1.0×10^4^ preosteoblasts were seeded on HAp-coated glass substrates (n=3) and cultured for one day to evaluate the toxicity of materials. Cells were washed with 1x PBS for 5 min and incubated with a 2 μM Calcein acetoxymethyl (Calcein AM) and 4 μM Ethidium homodimer-1 (EthD-1) mixture for 15 min at 37°C in darkness. Cells were washed with 1x PBS for 5 min and images were acquired using a fluorescence microscope (CKX41-F32FL; Olympus).

### 2.4 Cell morphology and focal adhesion (FA)

The morphology and focal adhesion of cells were investigated by spin coating the HAp samples on glass substrates; 1.0×10^4^ preosteoblasts were cultured using the above mentioned growth medium for one day on HAp- coated substrates. The cells were fixed using 4% paraformaldehyde, washed in 1x PBS, dehydrated in a graded ethanol solution (50%, 75% and 100%), completely dried, sputter-coated with platinum and then examined using SEM (Phenom G2 pro desktop, Eindhoven, Netherlands). Fluorescence staining of F-actin (CSK), FA molecules (vinculin) and cell nucleus was performed in order to examine the FA assembly pattern on HAp-coated substrates. The one-day cultured cells on HAp-coated glass substrates were rinsed twice with a PBS buffer, permeabilized with 0.1% Triton X-100 and washed again. They were then incubated for 30 min at room temperature with a 1% BSA blocking agent and washed twice with a PBS buffer. Mouse monoclonal anti-vinculin (0.67 μg/mL) was added to the cells, incubated for 60 min at 37°C and then washed three times in PBS. Fluorescein isothiocyanate (FITC)-conjugated goat anti-mouse IgG (10 μg/mL) from Chemicon (Chemicon International, Temecula, CA) and tetramethylrhodamine isothiocyanate (TRITC)-conjugated phalloidin (37.5 ng/mL) were loaded and incubated for 60 min at RT. The cells were washed three times, incubated for 5 min with a 4, 6-diamidino-2-phenylindole (DAPI) solution, and then rinsed several times. Stained cells were kept in PBS at 4°C. The fluorescence-stained cells were subsequently viewed and photographed using a confocal microscope (Olympus FluoView FV1000, Tokyo, Japan) at x600 magnification. Three independent samples were examined for each group.

### 2.5 Statistical analysis

Analysis of the differences between groups for each time point was performed by one-way ANOVA, followed by Tukey's post-hoc test. All statistical analyses were performed using GraphPad Prism software (version 5.0), with α=0.05.

## 3. Results and discussion

### 3.1 Structural analysis

**Figure 1.**
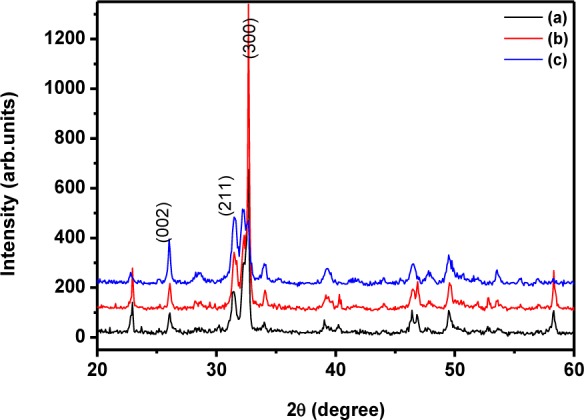
XRD pattern for the HAp nanoparticles prepared at different temperatures (a) 0°C; (b) room temperature; (c) 60°C

The phase purity and crystalline nature of the HAp nanostructures prepared at different temperatures were analysed using powder XRD. Figure 1 shows the XRD pattern for the as-prepared HAp nanostructured particles obtained at different temperatures.

**Figure 2.**
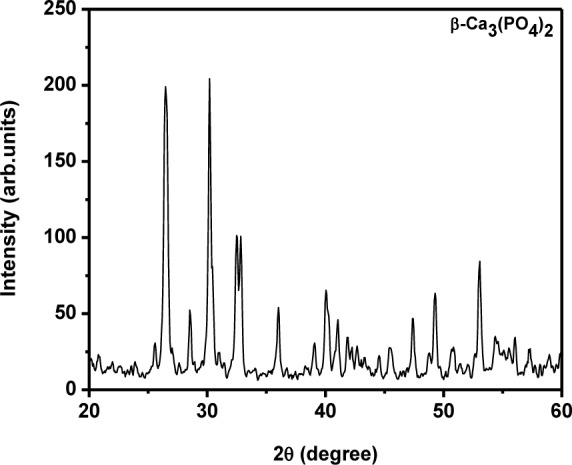
XRD pattern for the ß-tricalcium phosphate prepared at 90°C

The diffraction peaks in the XRD pattern matched the pure hexagonal phase [space group: P6_3_/m (176)] with the lattice parameters of *a*=9.418 Å and *c*=6.884 Å (JCPDS card # 09–0432) very well. The sample prepared at 60°C over 24 h had well-defined crystalline peaks (Figure 1c) with an average crystallite size of 42 nm. The size of the crystallite was small when compared to that of the sample prepared at room temperature (46 nm) and 0°C (50 nm). The average crystallite size was calculated from the XRD pattern using the Scherrer formula. The mean crystallite size along the *c*-axis (dimension) of the HAp crystals was 45 nm. Interestingly, the samples prepared at 0°C and room temperature lead to preferential growth and showed a rod-like morphology [31]. It can clearly be observed in Figure 1a and b that the peak corresponding to the (300) plane had greater intensity than the (211) plane [31]. However, on increasing the temperature to 60°C, the intensity of the (300) plane became lower than that of the (211) plane, which represented the formation of granules due to the distraction of the nucleation process for the formation of rod-like structures. Meanwhile, an increase in temperature to 90°C led to a change of phase from HAp to ß-TCP (ß-tricalcium phosphate, Whitlockite), as shown in Figure 2; additionally, the obtained XRD peaks match the standard JCPDS card # 09–169 very well. The average crystallite size was estimated as 46 nm. The Ca/P stoichiometric ratio and the calcination temperature play significant roles in the change of phase from HAp to ß-TCP; in the present case, however, increasing the reaction temperature induced the phase transformation from HAp to ß-TCP. It has already been reported that reaction temperature plays a critical role in phase change from HAp to ß-TCP [32]. This may be due to the nanosizes of the HAp particles, which are sensitive to heat treatment. Thus, the present study confirmed the significant role of reaction temperature in determining the structure and crystallinity of the sample.

**Figure 3.**
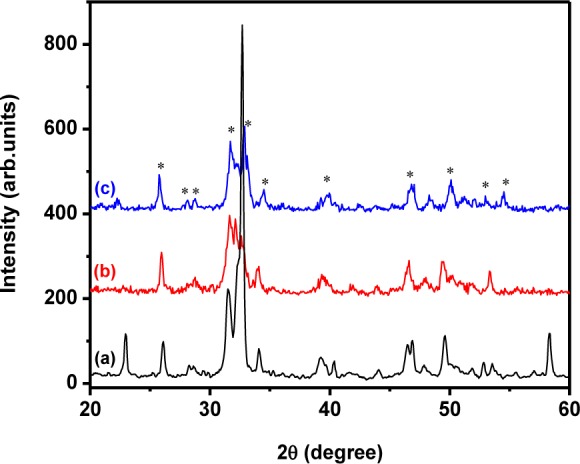
XRD pattern of the HAp nanoparticles prepared at different stirring durations: (a) 24 h; (b) 48 h; (c) 72 h

Figure 3 shows the XRD patterns for the pure HAp nanostructures prepared at different stirring times of 24, 48 and 72 h. It clearly depicts the crystalline structures of pure HAp. The reaction carried out at 24 h led to preferential growth along the *c*-axis. This was confirmed through the higher intensity of the (300) plane when compared to the (211) plane. Additionally, some other phosphate phases were observed in this pattern; this may have been due to the weak stirring effect [33]. The increase in stirring time to 48 h induced the particles' growth. Figure 3 clearly shows that the intensity of the (300) plane was less than that of the (211) plane and no other peaks were observed in this pattern. Further increase in the stirring time to 72 h led to peak broadening with less intensity, which showed poor crystallinity of the sample. Hence, it was observed that the 48 h stirring was suitable for producing highly crystalline HAp nanogranules. The average crystallite sizes of the HAp nanogranules were 46, 27, and 37 nm for 24, 48 and 72 h samples, respectively.

The formation of the hydroxyapatite can be explained using the following simple reactions (Eq. 1 and 2):

(1) $$\begin{array}{l} 10CaCl_{2}+6{\left(NH_{4}\right)}_{2}HPO_{4}+8NH_{4}OH\to \\ Ca_{10}{\left(PO_{4}\right)}_{6}{\left(OH\right)}_{2}+20NH_{4}Cl+6H_{2}O \end{array}$$

(2) $$Ca_{10}{\left({\text{PO}}_{4}\right)}_{6}{\left(\text{OH}\right)}_{2}\to 3Ca_{3}{\left({\text{PO}}_{4}\right)}_{2}+CaO+H_{2}O$$

**Figure 4.**
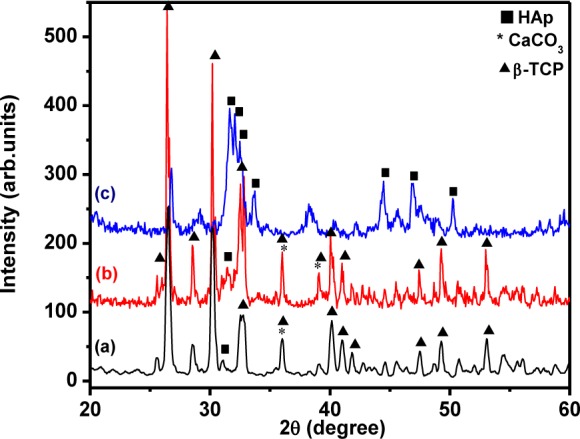
XRD pattern of HAp nanostructures prepared at different pH values: (a) 7; (b) 9; (c) 11.5

Figure 4 shows the XRD pattern for the HAp nanostructures prepared at different pH values and with a constant stirring time of 48 h. The formation of the HAp nanostructure was very weak when the pH values were 7 and 9. Also shown is the formation of other calcium phosphate phases like β-TCP and calcium carbonate [1, 32]. However, increasing the pH value to 11.5 induced the formation of pure HAp. The aqueous precipitation of HAp nanostructures based on CaCl_2_ and (NH_4_)_2_HPO_4_ provided a simple chemical reaction. Here, the precipitation of phosphate anions (PO_4_^3−^) into calcium ions (Ca^2+^) suspension was slow at lower pH values of 7 and 9 [34]; this led to the formation of B type carbonate at a minimum level during the formation of HAp, which is clearly shown in Figure 4 [35]. However, in the higher pH of 11.5 and using Ca(OH)_2_, H_3_PO_4_ as a starting precursor, the HAp was formed alongside CaO. This may have been due to the exhaustion of hydroxyl ions present in the Ca(OH)_2_ solution by H3PO_4_ [34]. In the present case, however, CaCl_2_ and (NH_4_)_2_HPO_4_ as starting precursors helped to avoid the formation of CaO during the change in pH. In order to enhance the precipitation of an anion to cation solution, a few drops of ammonia solution were added. Hence, the precipitation of PO_4_^3−^ ions with Ca^2+^ ions increased at a higher pH of 11.5. These results confirmed that the starting precursor material and pH played a significant role in obtaining pure HAp.

**Figure 5.**
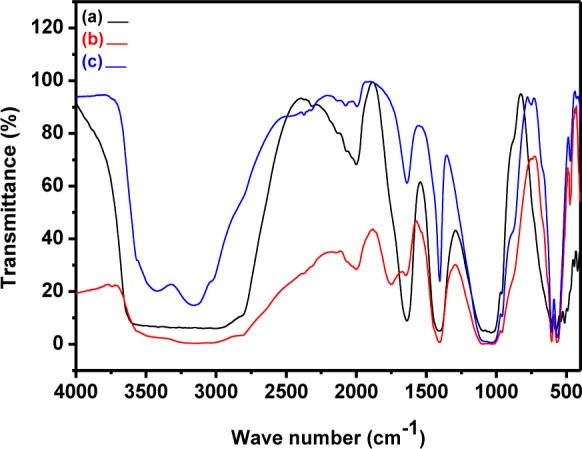
FTIR spectra of pristine HAp prepared at different temperatures: (a) 0°C; (b) room temperature; (c) 60°C

### 3.2 FTIR analysis

The presence of characteristic phosphate groups and surface hydroxyl groups were identified through FTIR spectroscopy. Figure 5 shows the FTIR spectra of the HAp prepared at different temperatures. In Figure 5, the sample prepared at 0°C shows broad and high intensity peaks at 2900 to 3700 cm^−1^. The peak observed at 1639 cm^−1^ was due to the reflection by absorbed and combined water. The peak at 610 cm^−1^ represents the OH^−^ vibrational mode. The characteristic PO_4_^3−^ peaks were observed at 1031 (ν_3_), 962 (ν_1_), 610 (ν_4_) and 575 (ν_4_) cm^−1^. Furthermore, a larger peak at 1408 cm^−1^ and smaller peak at 874 cm^−1^ was attributed to the CO_3_^2−^ group and may have been due to the presence of carbon dioxide during synthesis. This further indicates the substitution of CO_3_^2−^ for the PO_4_^3−^ position in the HAp lattice (B-type carbonate) [35–37].

Similarly, the sample prepared at room temperature showed surface hydroxyl group and phosphate group peaks at the standard positions. The peak observed at 3572 cm^−1^ represents the vibrational modes of the structural OH^−^ and the adsorbed H_2_O peak was seen at 1648 cm^−1^. The 3572 cm^−1^ peak was very weak due to an overlap with the broad strong peak of adsorbed water molecules. Tetrahedral PO_4_^3−^ bands were clearly observed at 475, 569, 606, 968 and 1021 cm^−1^. The peaks at 968, 475, 1021, 569 and 606 were attributed to PO_4_^3−^ group vibrations such as ν_1;_ ν_2_, ν_3_ and ν_4_ respectively. The CO_3_^2−^ peaks observed at 1406 and 874 cm^−1^ resulted due to interaction with atmospheric air. Moreover, it also revealed the existence of the carbonate group [38–40].

The FTIR spectrum for the sample prepared at 60°C is shown in Figure 5c. A very weak peak observed at 3572 cm^−1^ was attributed to the overlapping of the structural OH groups of HAp with broad H_2_O peaks. The peaks at 3423 and 1639 cm^−1^ revealed the presence of H_2_O molecules. The phosphate peaks were seen at 473, 564, 605, 959 and 1021 cm^−1^. The peaks at 1403 and 876 cm^−1^ represent the carbonate group positions in the HAp lattice [36, 41]. However, the percentage of CO_3_^2−^ varied in the samples prepared at 0°C, room temperature and 60°C, as observed in (Figure 5).

**Figure 6.**
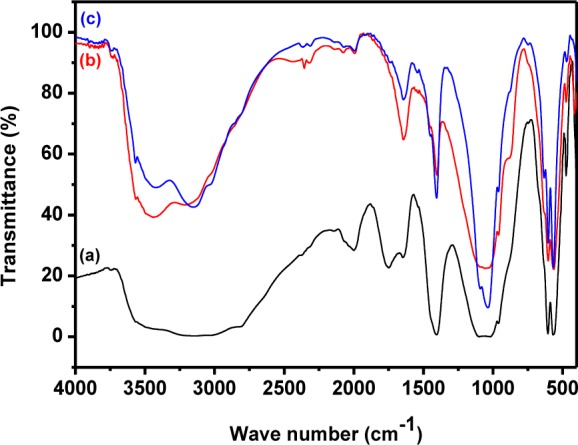
FTIR spectra of pure HAp prepared at different times: (a) 24 h; (b) 48 h; (c) 72 h

Similarly, the FTIR spectra of pure HAp nanostructures prepared at different stirring times are shown in Figure 6. In these spectra, all the structural hydroxyl, phosphate peaks and carbonate peaks were observed at the standard positions [35, 42].

### 3.3 Raman analysis

**Figure 7.**
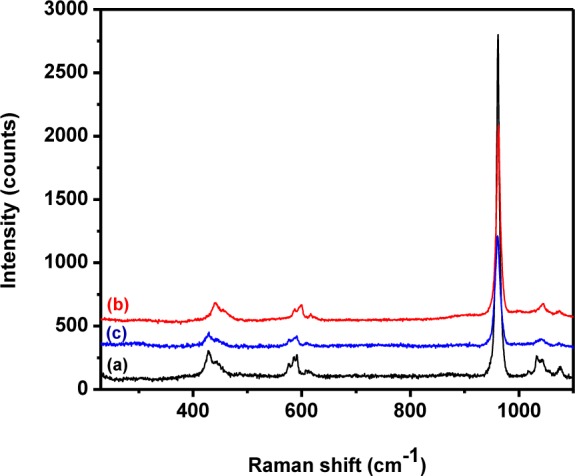
Raman spectra of pure HAp prepared at different temperatures: (a) 0°C; (b) room temperature; (c) 60°C

**Figure 8.**
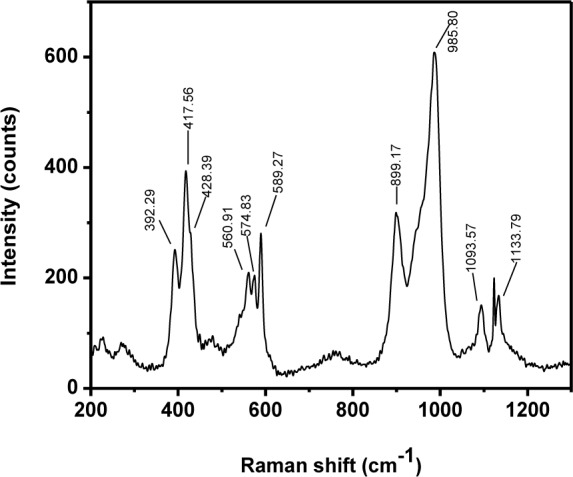
Raman spectrum of ß-TCP nanorods

Raman spectra of the HAp samples prepared at different temperatures are shown in Figure 7. All the peaks appeared at the standard positions [42]. The peak at 1043–1045 cm^−1^ (ν_3_) was attributed to triply degenerate asymmetric ν_3_ (PO) stretching. The tetrahedral PO_4_^3−^ internal mode (ν_1_) observed at 963 cm^−1^ represents the symmetric stretching of the P-O bond. The position of this peak represents the degree of crystallinity of the material [11] and confirmed that the prepared HAp had a more ordered and highly crystalline nature. Furthermore, the FWHM of the peaks at 962 cm^−1^ decreased when the preparation temperature was raised from 0° to 60°C and this was due to the reduction in particle size at higher temperatures [44]. The peaks at 588, 599 and 608 cm^−1^ revealed the triply degenerate bending mode of ν_4_ PO_4_^3−^ and the 440–444 cm^−1^ peak represented the double degenerate bending mode of the O-P-O bond of the (ν_2_) PO_4_^3−^ group. The peak at 1075 cm^−1^ confirmed the B-type CO_3_^2−^ present in the HAp [11]. The other carbonate modes such as ν_2_, ν_3_ and ν_4_ had weak intensities and were not detected [42]. Figure 8 shows the Raman spectrum of ß-tricalcium phosphate nanorods, which is akin to previously reported results [43].

**Figure 9.**
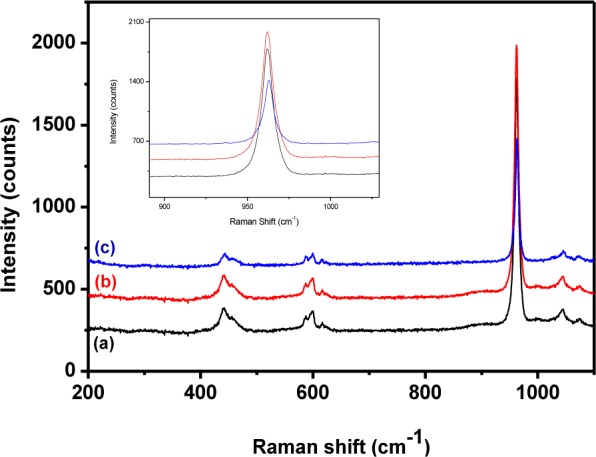
Raman spectra of pure HAp prepared at different stirring times: (a) 24 h; (b) 48 h; (c) 72 h

Figure 9 shows the typical Raman spectra of pristine HAp at different stirring durations. All the obtained peaks matched with those found in previous reports [11, 42]. A weak peak observed at 1074–1076 cm^−1^ represented the B-type carbonate (ν_3_) present in the sample. The intensity of the peak was observed to decrease with an increase in stirring. The FTIR and Raman results confirmed that the pristine HAp had good crystalline nature and structural integrity. It further confirmed the occupancy of the phosphate positions in the HAp lattice by the carbonate group.

### 3.4 Morphological analysis

**Figure 10.**
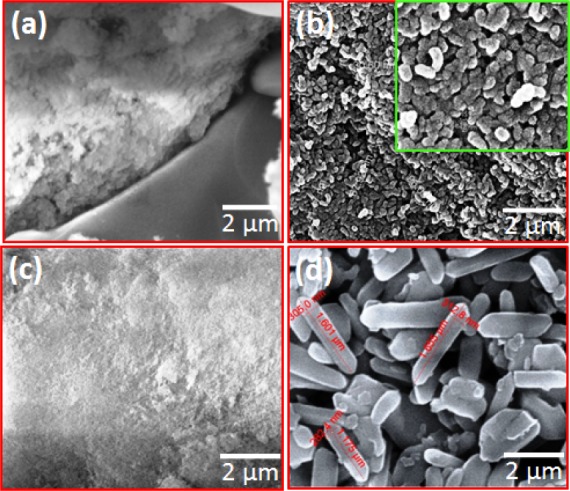
SEM images of pure HAp nanoparticles prepared at different temperatures: (a) below room temperature (0°C); (b) room temperature; (c) 60°C; (d) 90°C. The inset (b) was set at a higher magnification resolution

Figure 10 shows the scanning electron microscopic (SEM) images of the as-prepared HAp obtained at 0°C, room temperature and 60°C, with a pH value of 11.5 and stirring time of 24 h. Figure 10a shows the agglomeration of the particles as a result of the incomplete reaction at 0°C. Interestingly, the sample prepared at room temperature showed a bacteria-like morphology composed of spherical particles with an average size of 50 nm, as seen in Figure 10b. Increasing the temperature to 60°C led to an agglomeration of particles, as seen in Figure 10c. This may be due to the Ostwald ripening process and capillary force. However, a further increase in the reaction temperature to 90°C brought notable changes in morphology and phases, as seen in Figure 10d. The higher temperature broke the Ostwald ripening and capillary force and induced nucleation along the *c*-axis to form nanorods. The existence of such β-TCP nanorods had already been confirmed by XRD analysis. The average diameter of the rod was ∼300 nm and length was ∼1.5 μm. The aspect ratio (i.e., length/width) of the prepared β-TCP nanorods was ∼5 [28, 29].

**Figure 11.**
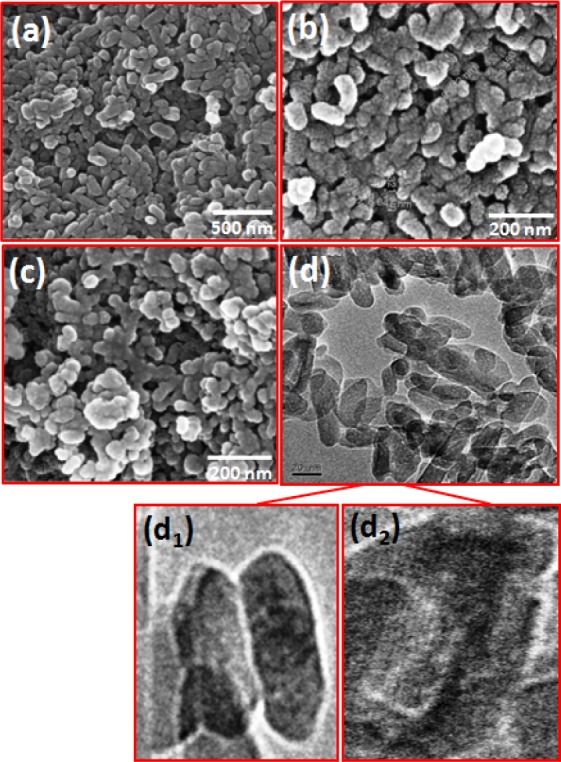
FESEM images of hydroxyapatite nanostructures prepared at different stirring times: (a) 24; (b) 48; (c) 72 h and a TEM image of optimized HAp (d); (d_1;_ d_2_) shows higher magnification image

Figure 11 shows the FESEM images of HAp samples prepared at different stirring times of 24, 48 and 72 h with a pH of 11.5 at room temperature. The bacteria-like HAp nanostructures seen in Figure 11a consisted of spherical particles with a size of ∼50 nm. The formation rate of HAp from other CaPs phases was slow. The samples prepared with a stirring time of 48 h had nanogranules as seen in Figure 11b. The average granule size was ∼10–15 nm and a reduction in particle size was due to the higher stirring time. TEM was employed for an optimized condition sample (room temperature, 48 h with pH 11.5) and the TEM image is shown in Figure 11d. This image confirmed that the optimized sample had a bacteria-like nanostructure. The size of granules was below 15 nm. When the stirring time increased to 72 h, solid agglomeration occurred, as shown in Figure 11c. The excess stirring energy may lead to high agglomeration with an irregular and bigger particle size.

**Figure 12.**
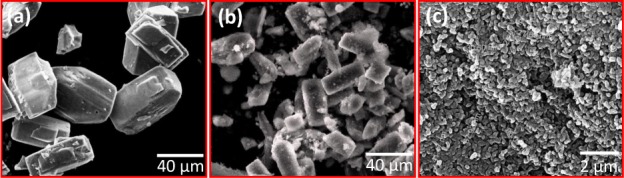
FESEM images of hydroxyapatite nanostructures prepared with different pH levels: (a) 7; (b) 9; (c) 11

A mixture of calcium phosphate phases was observed for the HAp prepared at pH values of 7 and 9. The reactions were carried out at room temperature for 48 h. Micro- sized rectangular crystals were observed in the sample prepared at a pH of 7, which was composed of CaCO_3_ and β-TCP with traces of HAp, as seen in Figure 12a. On further increasing the pH to 9, the rectangular crystal structure was transformed to a sponge-like structure, as observed in Figure 12b. This structure also consisted of CaCO_3_, β-TCP and traces of HAp. In both cases, the pH value was less and therefore, the formation rate of HAp was very poor [1, 32]. The samples prepared with a pH value of 11.5 showed immature particles grown along one direction, as well as an increased rate of HAp formation. The HAp granules joined together to form bacteria-like nanostructures. Their average diameter was 25–40 nm and length was ∼70–90 nm. Other calcium phosphate phases were not observed in these samples. The alkaline medium induced the nucleation of the calcium phosphate phase over the hydroxyapatite phase. Due to the existence of a number of phosphate compounds, this calcium phosphate system was denoted as the most complex family of materials. Moreover, the compositional changes and reaction conditions such as precursors, temperature, time, pH, solvent, pressure and ligand all played a pivotal role in the stability of different phosphate phases [29, 45].

The typical experimental parameters and corresponding FESEM results of the hydroxyapatite nanostructures prepared with different stirring durations and with different pH values are listed in Table 1.

**Figure 13.**
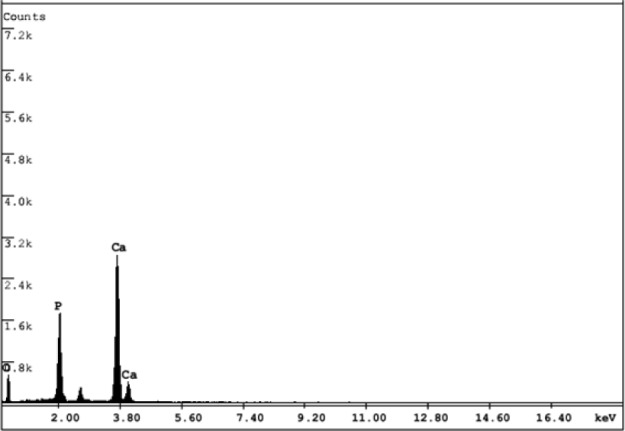
EDX spectrum of the HAp

Figure 13 shows the typical EDX spectrum of the pristine HAp prepared at room temperature with a reaction time of 48 h and a pH of 11.5. Similar spectra were obtained for all other samples prepared under different conditions. The EDX analysis confirmed the composition of pristine hydroxyapatite, as well as the occurrence of Ca, P and O. The results showed no other elements or impurities in the sample.

**Table 1.** The experimental parameters and representative results obtained under different reaction conditions

S.No	Temperature $\left({\;}^{\underline{o}}C\right)$	Stirring time (h)	pH	Product morphology
1	0	24	11.5	Incomplete reaction, badly defined particles
2	27	24	11.5	Bacteria-like nanostructure composed of non-uniform particles
3	60	24	11.5	Deposition like nanoparticles
4	90	24	11.5	β-TCP nanorods
5	27	24	11.5	Bacteria-like nanostructure composed by non-uniform particles
6	27	48	11.5	Bacteria-like nanostructure composed by more uniform nanogranules
7	27	72	11.5	Bacteria-like nanostructure made up of agglomerated particles
8	27	48	7.0	Micro-sized rectangle composed by CaCO_3_, β-TCP with traces of HAp
9	27	48	9.0	Spongy-like micro-sized rectangle composed of CaCO_3_, β-TCP, with traces of HAp
10	27	48	11.5	Bacteria-like nanostructure composed of more uniform nanogranules

**Figure 14.**
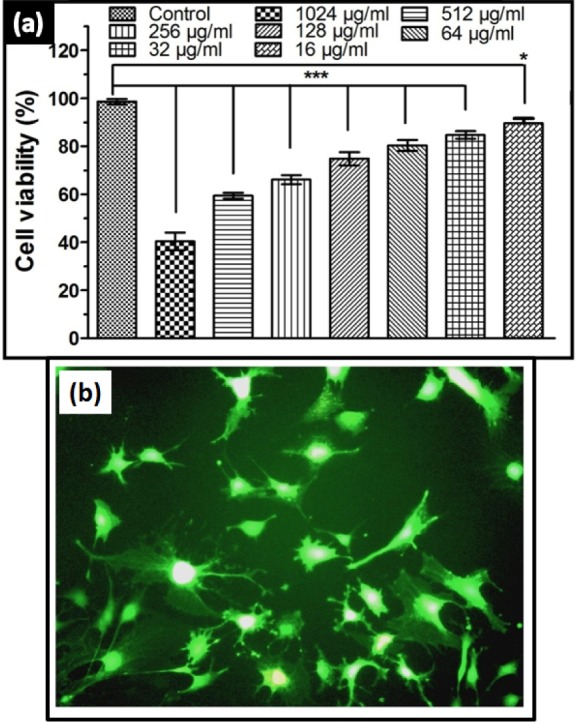
(a) Cell viability of bacteria-like HAp nanostructures at different concentrations prepared at 48 h at room temperature; (b) Fluorescent microscopic image for cell viability of HAp nanostructures on preosteoblast cells

### 3.5 In-vitro cell viability, proliferation, morphology and focal adhesion

In the present work, bacteria-like HAp nanostructures (prepared at room temperature, 48 h stirring and a pH of 11.5) were exclusively used for in vitro evaluation. Figure 14a shows the viability of preosteoblasts treated with HAp. The serially diluted samples were used for the CCK-8 assay and the obtained result is given in Figure 14a. The viability of the cells on day 1 was above 80% for the HAp samples with a concentration lower than 128 μg/ml; however, negligible cytotoxicity was observed. Conversely, decreased cell viability (40.4%) was noticed alongside an increase in HAp concentration (1024 μg/ml), due to the agglomeration of HAp nanostructures into larger particle sizes, which causes low crystallinity. This lower crystallinity in turn led to increased dissolution and solubility, which was assumed to be the reason for the cell membrane rupture [46]. The surface area of the HAp nanostructures decreased upon increasing the concentration and this played a key role in the proliferation and adhesion of the cells [47]. The fluorescent microscopic image in Figure 14b depicts live cells (green colour); no dead cells were found, which evidenced the excellent cell viability of preosteoblasts on HAp nanostructures. The strong deposition of HAp on any given substrates reduced the freely available HAp in the culture medium and decreased the cell-HAp material interaction, resulting in increased cell viability. Additionally, the size and surface area of the HAp nanostructure played a major role in determining cell adhesion properties [36].

**Figure 15.**
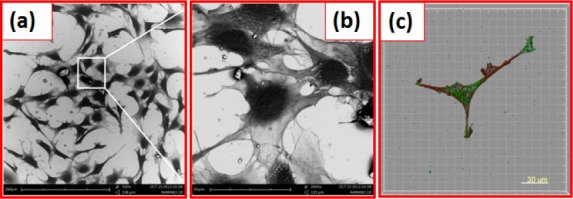
SEM images of (a) HAp nanostructures on osteoblast cells; (b) higher magnification image; (c) focal adhesion of cells

The bacteria-like HAp nanostructure consisted of fine and uniform size granules and as a result, cell adhesion was higher on the surface, as confirmed by SEM images shown in Figure 15a. The cells were actively proliferated and spread well on the highly crystalline bacteria-like HAp nanostructure, with active filopodia interactions indicating extensive cell migration and cell-cell interaction, as shown in Figure 15b. This cell behaviour demonstrated the osteoconductive nature of the HAp nanostructures. Additionally, fluorescent staining of the cytoskeleton (F-actin), an FA molecule (vinculin) and the cell nucleus was performed in order to examine the FA assembly pattern on HAp nanostructure substrates, as shown in Figure 15c. Vinculin expression of preosteoblasts on HAp nanostructures was strong both in the periphery and in the centre of cells. F-actin staining evidenced the presence of wide-spread cytoskeleton cells on HAp substrates. Overall, in vitro results indicated that bacteria-like HAp nanotopography offers a promising and better surface platform for preosteoblast adhesion, migration and proliferation.

## 4. Conclusion

In summary, pristine HAp nanostructures were synthesized by a simple, cost effective and surfactant free chemical precipitation method using CaCl_2_ and (NH_4_)_2_HPO_4_ as starting precursors. The present study describes the crystallinity and morphological changes of HAp nanostructures with respect to different preparation conditions such as reaction temperature, stirring time and reaction pH. The HAp prepared at room temperature with 48 h stirring and a 11.5 pH value showed a well- defined size and morphology, better than the HAp nanostructures prepared under other conditions. Furthermore, an increment in reaction temperature to 90°C brought a change of phase from HAp to β-TCP and morphology changed from particles to nanorods. The results obtained from all the analytical studies confirmed that the physicochemical nature of the HAp strongly depended on the reaction parameters. The biocompatibility and adhesion nature of the prepared HAp nanostructures were investigated using a mouse preosteoblast cell line through an in vitro method. The obtained results proved the excellent viability, adhesion and proliferation of the prepared HAp nanostructures.
